# Fatal Hæmorrhage from Varix of the Vulva

**Published:** 1844-12

**Authors:** 


*Fatal Hæmorrhage from a Varix of the Vulva.*—In a late number of the Berlin Medical Gazette, we find the following case related:

A woman, pregnant of her fifth child, had, for a length of time, been annoyed with a large swelling on the left labium. One evening, immediately after going to bed, she was suddenly seized with a profuse hæmorrhage from the vulva. The midwife was accordingly summoned to her assistance; but she. finding the alarming state of the woman, sent off at once for Dr. Hesse. On his arrival, the patient was already in the agonies of death: the bed and floor were covered with dark coagulated blood. On examination, the orifice of the vagina was found closed, and its canal quite dry. The Cæsarian operation was immediately performed; but the child was dead. The uterus was quite empty of blood; and the placenta adhered over its entire extent. No distinct tumour was now visible on the vulva, although the labium was still flabby and voluminous. When it was pressed, a jet of dark blood flowed out from a wound that was not more than half an inch in extent. This wound led backwards, in the direction of the perineum, to a cluster of varicose veins, one of which had burst.—*Med. Chir. Review.*

